# An *In Vitro* ES Cell-Based Clock Recapitulation Assay Model Identifies CK2α as an Endogenous Clock Regulator

**DOI:** 10.1371/journal.pone.0067241

**Published:** 2013-06-28

**Authors:** Yasuhiro Umemura, Junko Yoshida, Masashi Wada, Yoshiki Tsuchiya, Yoichi Minami, Hitomi Watanabe, Gen Kondoh, Junji Takeda, Hitoshi Inokawa, Kyoji Horie, Kazuhiro Yagita

**Affiliations:** 1 Department of Physiology and Systems Bioscience, Kyoto Prefectural University of Medicine, Kyoto, Japan; 2 Department of Social and Environmental Medicine, Osaka University Graduate School of Medicine, Osaka, Japan; 3 Laboratory of Animal Experiments for Regeneration, Institute for Frontier Medical Sciences, Kyoto University, Kyoto, Japan; 4 Precursory Research for Embryonic Science and Technology, Japan Science and Technology Agency, Saitama, Japan; 5 Department of Physiology II, Nara Medical University, Nara, Japan; University of Texas Southwestern Medical Center, United States of America

## Abstract

We previously reported emergence and disappearance of circadian molecular oscillations during differentiation of mouse embryonic stem (ES) cells and reprogramming of differentiated cells, respectively. Here we present a robust and stringent *in vitro* circadian clock formation assay that recapitulates *in vivo* circadian phenotypes. This assay system first confirmed that a mutant ES cell line lacking *Casein Kinase I delta* (*CKIδ*) induced ∼3 hours longer period-length of circadian rhythm than the wild type, which was compatible with recently reported results using *CKIδ* null mice. In addition, this assay system also revealed that a *Casein Kinase 2 alpha* subunit (*CK2α*) homozygous mutant ES cell line developed significantly longer (about 2.5 hours) periods of circadian clock oscillations after *in vitro* or *in vivo* differentiation. Moreover, revertant ES cell lines in which mutagenic vector sequences were deleted showed nearly wild type periods after differentiation, indicating that the abnormal circadian period of the mutant ES cell line originated from the mutation in the *CK2α* gene. Since *CK2α* deficient mice are embryonic lethal, this *in vitro* assay system represents the genetic evidence showing an essential role of *CK2α* in the mammalian circadian clock. This assay was successfully applied for the phenotype analysis of homozygous mutant ES cells, demonstrating that an ES cell-based *in vitro* assay is available for circadian genetic screening.

## Introduction

The circadian clock is an intrinsic time-keeping system regulating various physiological functions such as sleep/awake cycle, body temperature and metabolism [1]–[3]. The core component is the cell-autonomous molecular oscillator comprised of transcriptional-translational feedback loops of clock genes such as *Bmal1*, *Clock*, *Period* (*Per1, 2, 3*) and *Cryptochrome* (*Cry1, 2*) [1]. Two transcription factors CLOCK and BMAL1 transactivate the *Per* genes, *Cry* genes and *Rev-Erbα* via the E-box enhancer elements. Expressed PER and CRY then suppress CLOCK/BMAL1 activity, which results in the cyclic activation of these clock genes [1], [4], [5]. The *Bmal1* gene also shows cyclic expression but an anti-phasic pattern with E-box driven clock genes because of REV-ERBα cyclically activate the *Bmal1* transcription [6]. In these circadian feedback loops, Casein Kinase I δ/ε (CKIδ/ε) have been known essential central kinases to regulate the stability of PER proteins through their phosphorylation [7]–[10].

It has been reported that the master pacemaker in the suprachiasmatic nucleus (SCN) develops in the late embryonic stage, and circadian rhythms subsequently appear around birth [11], [12]. Recently, our studies using mouse embryonic stem (ES) cells and *in vitro* differentiation culture suggested cell-autonomous development of circadian molecular oscillators in mouse ES cells during differentiation [13], [14]. ES cells showed no apparent molecular oscillation, in contrast to somatic cells. However, the circadian oscillation of clock gene reporters became detectable following *in vitro* differentiation. Moreover, reprogramming of differentiated, rhythmic cells into pluripotent stem cells resulted in the loss of circadian oscillation [13]. These results are consistent with the notion that cell-autonomous development of the mammalian circadian clock is coupled with cellular differentiation.

Genetic screening for circadian clock genes has been successfully conducted in mice using chemical mutagenesis [15], [16]. Our finding of *in vitro* circadian clock formation through ES cell differentiation provides us with the opportunity to develop a complementary screening system in tissue culture. We recently constructed a homozygous mutant ES cell bank which facilitates phenotypic analysis of various genes in tissue culture [17].

In the present study, we established a highly consistent differentiation protocol and conducted genetic analysis of circadian rhythm using our mutant ES cells. It has been revealed that CKIδ is essential as a central kinase of the mammalian circadian clock [7], [8], and that genetic ablation of *CKIδ* results in the lengthening of the circadian period for ∼2 hours in mouse embryonic fibroblasts and suprachiasmatic nucleus [18], [19]. In this study, we first tested the reliability of our *in vitro* circadian clock formation assay to see whether the definitive features of circadian clock such as temperature compensation and genetically determined phenotypes were correctly recapitulated using wild type ES cell line and homozygous mutant ES cell line lacking *CKIδ* expression.

In addition to CKIδ/ε, Casein Kinase 2 (CK2) has recently also been implicated in circadian clock regulation using genome-wide RNAi screening studies [8], [20]. In species other than mammals, CK2 has been revealed to play an essential role for circadian rhythm maintenance [21], [22]. However, detailed genetic analysis of *CK2* has been hampered in mammals by embryonic lethality in *CK2* knockout mice. We therefore chose *CK2α* homozygous mutant ES cell line from the homozygous mutant ES cell bank [17] and investigated the effect of *CK2α* deficiency on circadian rhythm.

## Materials and Methods

### Ethics Statement

All procedures with animals were approved by Kyoto Prefectural University of Medicine Animal Care Committee.

### Mutant ES Cells

Mutant ES cell lines for casein kinase I delta (abbreviated as *CKIδ* or *Csnk1d*) and casein kinase 2 alpha subunit (abbreviated as *CK2α* or *Csnk2a1*) were generated by insertional mutagenesis with the retroviral vector as described previously [17]. The vector insertion sites are as follows (mouse genome database mm9, July 2007): *CKIδ*: chromosome 11, position 12,0852,242; *CK2α*: chromosome 2, position 152,053,325.

### Cell Culture

Wild type ES cells, genetically mutated ES cell lines (*CKIδ* or *CK2α*), and their revertant ES cells [17] were used for *in vitro* differentiation. These ES cells were cultured on the feeder layer of mitomycin C-treated primary mouse embryonic fibroblasts in ES cell medium (ESM), which contains Glasgow Minimum Essential Medium (G-MEM, Wako) supplemented with 15% fetal bovine serum (FBS, Hyclone), 0.1 mM MEM nonessential amino acids (Nacalai Tesque), 0.1 mM 2-mercaptoethanol (Sigma), 1,000 units/mL of leukemia inhibitory factor (LIF), and 100 units/mL of penicillin–streptomycin (Nacalai Tesque).

To establish ES cells stably expressing *Bmal1:luc* reporter, 3 µg of *Bmal1:luc*-pT2A plasmid with Zeocin™ selection marker [13] and 1 µg of a Tol2 transposase expression vector (pCAGGS-TP) [23] were diluted in 35 µL of ESM and 12 µL of Fugene 6 transfection reagent (Promega) and mixed well. After a 15-min incubation at room temperature, the mixture was added to 2.5×10^5^ ES cells. The cells were selected with 100 µg/mL Zeocin™ (Invitrogen).

### *In vitro* Differentiation

**Figure 1:**
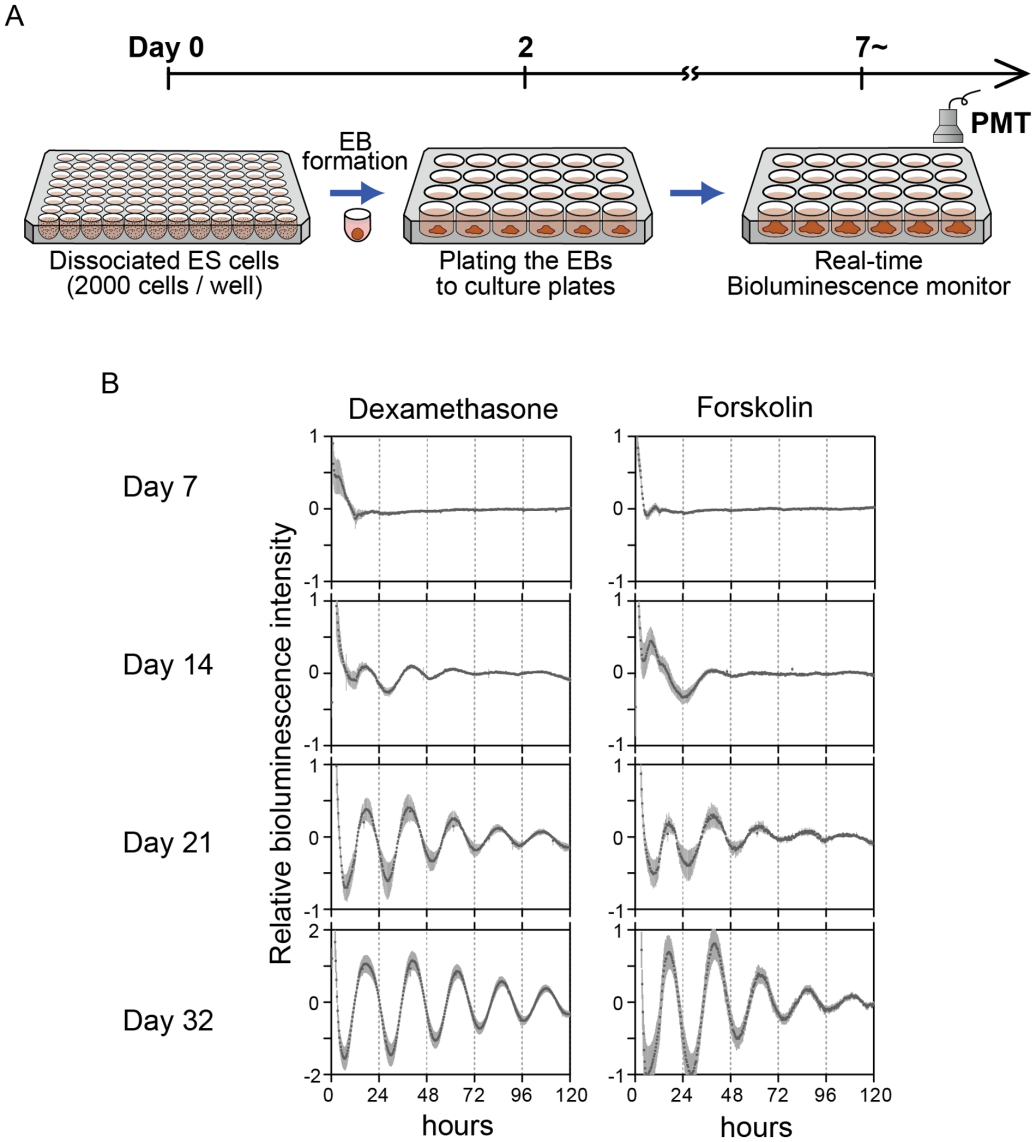
Establishment of *in vitro* circadian clock formation assay system using ES cells. (A) Scheme of the method for developing circadian oscillation *in vitro* via formation of embryoid bodies (EBs). EBs were generated from 2,000 ES cells and were seeded onto low-attachment 96-well plates in differentiating medium without LIF supplementation (see Methods). Two days later, EBs were plated onto gelatin-coated tissue culture 24-well plates (putting one ES onto one well) and cultured for several weeks. Subsequently, bioluminescence in each well was monitored by using PMT-based photon counting. (B) Averaged bioluminescence traces after *in vitro* 7, 14, 21, or 32-day differentiation of ES cells carrying *Bmal1:luc* reporter (*left*, Dexamethasone reset; *right*, Forskolin reset). Data detrended by subtracting a 24-h moving average are means with standard deviation (*n* = 24).

After ES cells were trypsinized, feeder cells were removed by incubating the cell suspension on a gelatin-coated 35-mm or 60-mm culture dish for 20 min at 37°C with 5% CO_2_. Embryoid bodies (EBs) were generated by harvesting the 2,000 cells and seeding them onto low-attachment 96-well plates (Lipidure Coat, NOF) in differentiating medium without LIF supplementation (EFM), that is high glucose Dulbecco’s modified Eagle medium (DMEM, Nacalai Tesque) containing 12% FBS, 1 mM sodium pyruvate (Nacalai Tescque), 0.1 mM nonessential amino acids, GlutaMax™-I (Invitrogen), 0.1 mM 2-mercaptoethanol (Sigma), and 100 units/mL penicillin-streptomycin. Two days later, EBs were plated onto gelatin-coated tissue culture 24-well plates (put one EB into one well) and grown for several additional weeks **(see** **Fig. 1A** **)**.

### Preparation of MEFs Derived from Chimeric Embryo

Chimeric embryos were generated from homozygous *CKIδ* or *CK2α* mutant ES cell lines and their parental (wild type) ES cell line by injection into C57BL/6 x DBA/2 F1 hybrid blastocysts. Chimeric embryos were collected at E13.5. After removal of the heads and visceral tissues, the remaining bodies were washed in fresh PBS and minced and the isolated cells were maintained in EFM.

### Real-time Bioluminescence Analysis

For real-time bioluminescence analysis of the cells seeded in 24-well black plates, the medium was replaced with EFM containing 0.2 mM luciferin (Promega) and 10 mM HEPES without phenol red. Synchronization was performed using 100 nM of dexamethasone or 10 µM of forskolin for 1 hour. The plates were set on the turntable of house-made 24-PMT head type real-time monitoring equipment [24]. The bioluminescence from each well was counted for 1 minute in every 20 minutes.

### Data Analysis

Period lengths of bioluminescence rhythms were estimated by RAP software (CHURITSU, Nagoya, Japan) using the cosinor method and based on Fourier analysis, specific for circadian rhythms [25]. Strength of rhythmicity was defined by spectral analysis (FFT relative power) as the relative spectral power density at the peak within the range of 20–28 hr [26]. The FFT analysis was applied to the whole five days excluding the first 12 h of data. For raster plots, bioluminescence-intensity data were detrended by subtracting a 24-h moving average, normalized for amplitude, and then color coded with red (higher than average) and green (lower than average). Plots were constructed using TreeView [27].

### Statistical Analysis

Statistical differences were evaluated using one-way ANOVA followed by a Bonferroni post hoc test. All statistics were calculated using GraphPad Prism version 5.0 software.

### Isolation of Revertant Clones

Revertant clones were isolated by transfecting homozygous clones with a FLPo expression vector followed by PCR screening for recombination events as described previously [17]. The following PCR primers at the flanking regions of the vector insertion sites were used:

*Casein kinase 1δ* : 5′-tgc cat gga gct gag ggt cgg gaa cag gta-3′ and 5′-tgc ggg gat gcc gaa cgt cca ct-3′.

*Casein kinase 2α* : 5′-gag atg tgg tag aaa gag aaa ggt tg-3′ and 5′-cct gtc acc ttt tca caa tac ttc tt-3′.

### Quantitative RT-PCR

MEF feeder cells were removed by plating the culture on a gelatin-coated dish for 20 min and transferring unattached ES cells onto a fresh dish. ES cells were harvested in the Isogen reagent (Nippon Gene) and the total RNA was extracted according to the manufacturer’s instructions. Power SYBR Green PCR Master Mix (Applied Biosystems) was used for real time PCR. Transcription levels were determined in triplicate reactions after normalization to 18S ribosomal RNA. Quantitative RT-PCR analysis was performed with a StepOnePlus real-time PCR system (Applied Biosystems). The amplification protocol comprised an initial incubation at 95°C for 2 min and 40 cycles of 95°C for 30 s and 60°C for 30 s, followed by dissociation-curve analysis to confirm specificity. Primer sequences are shown below:

*Casein kinase 1δ* Forward 5′-atc gcc aag gct tct cct-3′.

*Casein kinase 1δ* Reverse 5′-cca cga gtg gct gga ttc-3′.

*Casein kinase 2α* Forward 5′-tca gca gcg cca ata tga-3′.

*Casein kinase 2α* Reverse 5′-acc tct gct cag gca tca-3′.

## Results

### *In vitro* ES Cell-based Circadian Clock Formation Assay

To evaluate the effect of the mutations on the *in vitro* development of the circadian clock in ES cells, we improved our ES cell differentiation protocol and established a method for robust, reproducible and stringent circadian clock formation. Briefly, we first cultured dissociated 2,000 ES cells for two days in round-bottom low-attachment 96-well plates to allow formation of the embryoid body (EB). We subsequently transferred one EB into one well of 24-well plates for differentiation **(** **Figure 1A** **)**. To monitor development of circadian oscillation, we used a wild-type (WT) ES cell line stably transfected with a *Bmal1:luc* reporter. Whereas no circadian oscillation of *Bmal1:luc* reporter bioluminescence was detected even after Dexamethasone (Dex) or Forskolin (FSK) synchronization stimuli in an undifferentiated state, weak circadian bioluminescence first appeared in cultures after 14 days but rapidly dampened **(** **Figure 1B** **)**. The bioluminescence oscillation become more robust on day 21 and reached maximum amplitude on day 32 **(** **Figure 1B** **)**. In addition, all examined samples represented robust circadian clock oscillation and stringent reproducibility for quantitative analysis **(Figure S1)**. Moreover, the induced rhythms showed temperature compensation **(Figure S2)**, indicating the canonical biological nature of the circadian clock.

### Casein Kinase 1δ and Casein Kinase 2 Homozygous Mutant ES Cells

**Figure 2:**
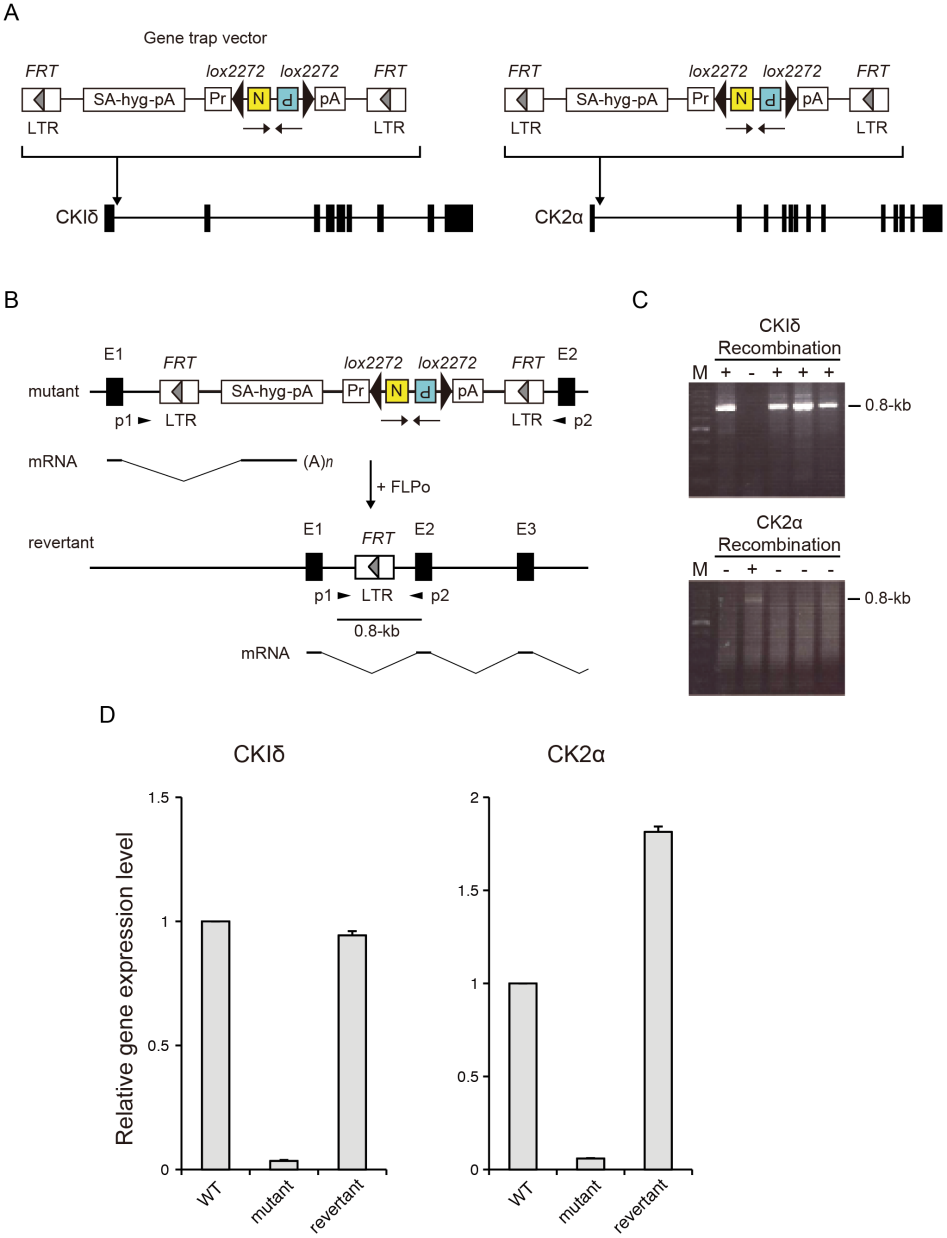
Characterization of *CKIδ* and *CK2α* homozygous mutant ES cell clones and their revertant clones. (A) Design of the gene trap vector and its insertion sites in *CKIδ* and *CK2α* homozygous mutant ES cell clones. SA, splice acceptor; hyg, hygromycin-resistance gene; pA, polyadenylation signal; Pr, phosphoglycerate kinase-1 promoter; N, neomycin-resistance gene; P, fusion gene comprised of the puromycin-resistance gene and the herpes simplex virus thymidine kinase gene; LTR, long terminal repeat. Horizontal arrows below the gene trap vector indicate orientation of the N and P drug resistance genes. (B) Schematic representation of the removal of mutagenic vector sequence after FLPo/FRT recombination. Recombination events were identified by PCR primers (p1 and p2) at the flanking region of the vector insertion sites. Removal of vector sequences regenerate wild type transcripts in the revertant allele. Note that the size of the gene trap vector, exons, introns are not to scale. E, exon. (C) PCR screening for FLPo/FRT recombination events. Note that PCR product was not detected in non-recombinant clones because of the large intervening vector sequence between primers. M, 100-bp DNA ladder. (D) Relative expression level of *CKIδ* and *CK2α* mRNA in wild type, mutant and revertant ES cells. Error bars show SEM (n = 3).

We recently constructed a homozygous mouse mutant ES cell bank using promoter trap vectors for insertional mutagenesis [17]. Currently, the bank has around 200 homozygous mutant ES cell lines and 2,000 heterozygous mutant ES cell lines. Database search of the mutant bank for the circadian period mutant identified two homozygous mutant ES cell lines, harboring mutation in *CKIδ* and *CK2α* respectively. We confirmed that homozygous mutation abolished expression of the *CKIδ* and *CK2α* gene **(** **Figure 2A and D** **).** As a control, we obtained revertant ES cell lines which regained *CKIδ* and *CK2α* expression respectively **(** **Figure 2B–D** **)** [**17**].

### Evaluation of Developed Circadian Clock Rhythmicity from Mutant ES Cell Lines

**Figure 3:**
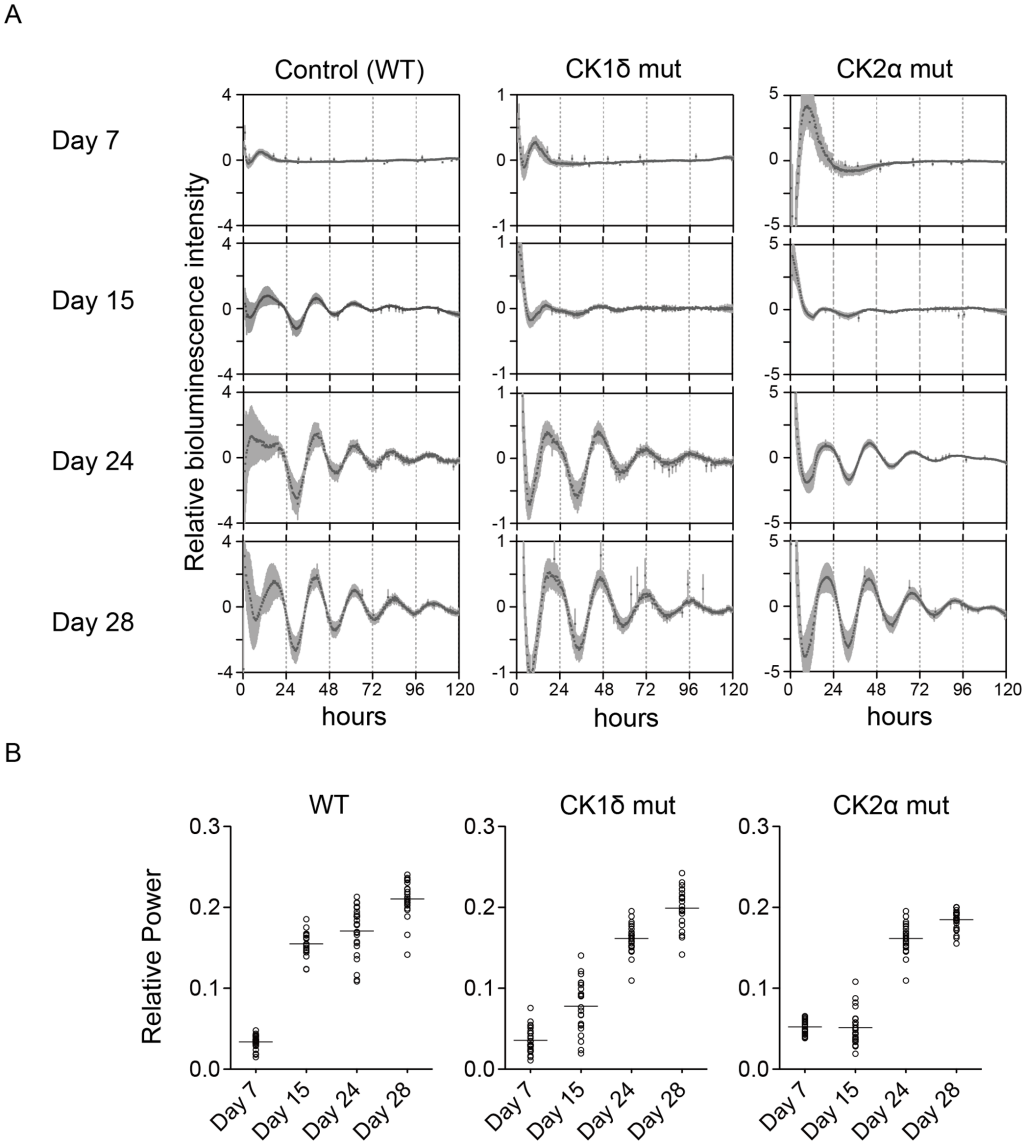
Development of mammalian circadian rhythm by using homozygous mutant ES cells. (A) Averaged bioluminescence traces after *in vitro* 7, 15, 24, or 28-day differentiation of ES cells carrying *Bmal1:luc* reporter (*left*, wild type ES cells (WT); *middle*, *CKIδ* mutant ES cells; *right*, *CK2α* mutant ES cells). Data detrended by subtracting a 24-h moving average are means with standard deviation (*n* = 24). (B) FFT spectral power analysis of bioluminescences of *in vitro* differentiated ES cells (7, 15, 24, or 28-day). Bars indicate mean values (*n* = 24). One circle represented *in vitro* differentiated ES cells from a single EB.

Using this *in vitro* differentiation culture method, *CKIδ* and *CK2α* homozygous mutant ES cells were differentiated *in vitro* and *Bmal1:luc* bioluminescence oscillation was observed. Similar to the WT ES cells **(** **Figure 3A left panel** **s)**, *CKIδ* and *CK2α* mutant ES cells developed circadian oscillation in a differentiation culture **(** **Figure 3A** **middle and right panels)**. Heat map plots **(Figure S3)** and quantitative Fast Fourier transformation (FFT) - relative power analysis **(** **Figure 3B left panel** **)** also indicated that circadian rhythmicity and amplitude developed progressively and reached the highest levels of power at around day 28 during the ES cell differentiation *in vitro*. These results suggest quantitative analysis of circadian clock formation would be possible after 28 days of differentiation.

**Figure 4:**
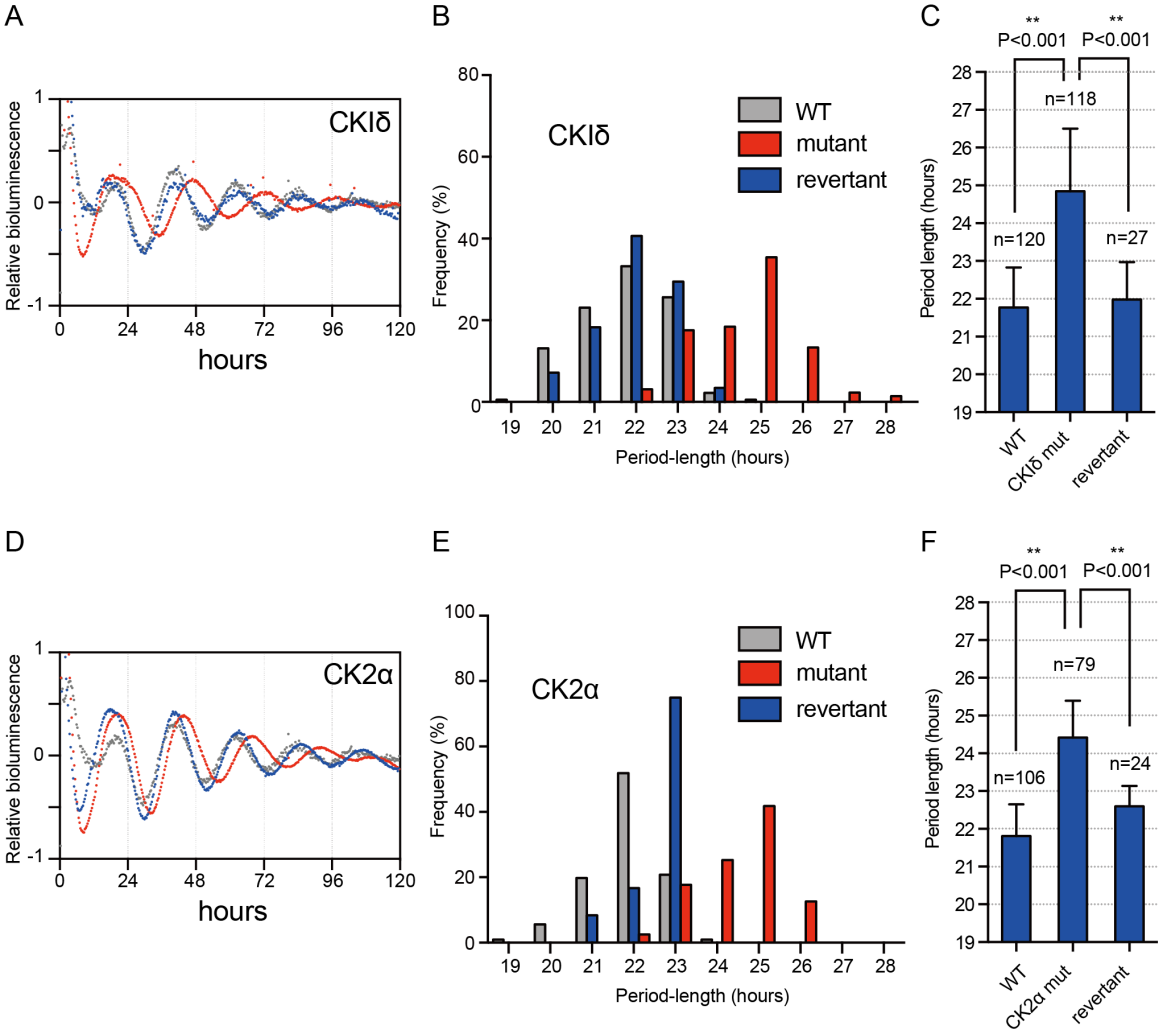
*In vitro* differentiated cells from *CKIδ* mutant and *CK2α* mutant ES cells show a longer period-length of the circadian clock than cells differentiated from wild type ES cells. After 28-day differentiation, the bioluminescence intensity in each well was monitored. (A, D) Averaged bioluminescence traces after *in vitro* 28-day differentiated *CKIδ* (A) or *CK2α* (D) mutant/revertant ES cells (*gray*, WT; *red*, homozygous mutant; *blue*, revertant). Data detrended by subtracting a 24-h moving average are means (*n* = 24). (B, C, E, F) Distributions and bar graphs of the period lengths of bioluminescence traces in each well. Error bars are SD. Statistical differences were evaluated using one-way ANOVA followed by Bonferroni post hoc test.

We next conducted in-depth analyses of the *CKIδ* and *CK2α* homozygous mutations on cellular circadian rhythmicity. After 28 days in the differentiation culture, bioluminescence monitoring was performed for five days. The results revealed that the *CKIδ* and *CK2α* deficient cells exhibited significantly lengthened circadian periods compared with WT and revertant cells **(** **Figure 4A and D** **)**. In addition, the period distribution of induced circadian clocks in *CKIδ* and *CK2α* mutants showed a slightly wider range than WT and revertant cells **(** **Figure 4B and E** **)**. The average period length of *CKIδ* and *CK2α* mutant cells were about 3.0 and 2.5 hours longer than that of WT, respectively **(** **Figure 4C and F** **)**. In contrast, revertant lines of both mutants showed WT-like period lengths **(** **Figure 4C and F** **)**.

### Genotype Dependent Effect on Circadian Period-length Observed in Mutant ES Cell-derived Embryonic Fibroblasts

**Figure 5:**
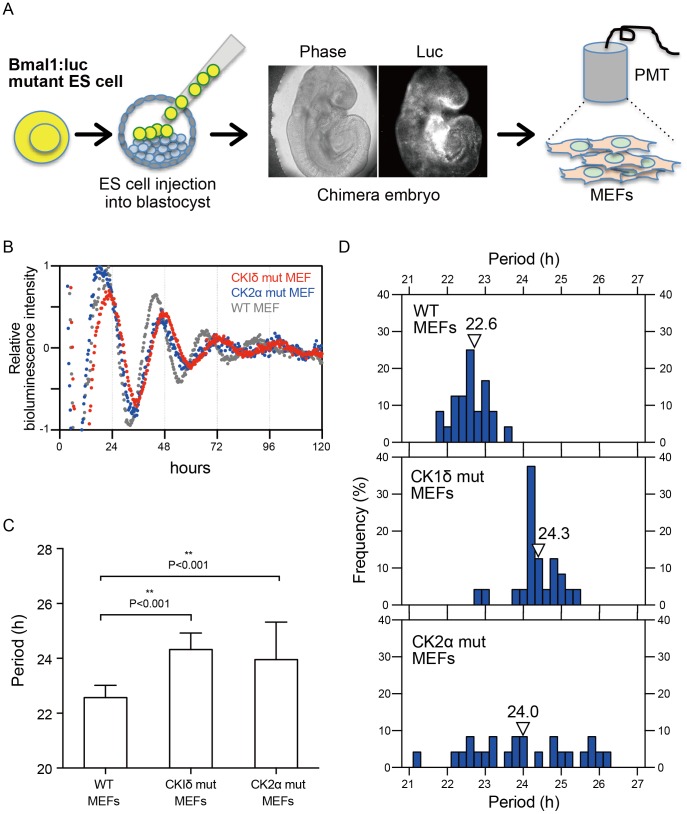
*CKIδ* and *CK2α* mutant MEFs developed *in vivo* show a longer period-length of circadian clock oscillation than wild type ES cell-derived MEFs. (A) Preparation of mouse embryonic fibroblasts (MEFs) from E13.5 chimera embryos. MEFs were maintained in EFM and their bioluminescence was monitored. Only MEFs derived from injected ES cells, not the host blastocyst-derived MEFs, contain *Bmal1:luc* reporter and produce bioluminescence. (B) Averaged bioluminescence traces of MEFs from *CKIδ* and *CK2α* mutant chimeric mice (*gray*, WT; *red*, *CKIδ* mutant MEFs; *blue*, *CK2α* mutant MEFs). Data detrended by subtracting a 24-h moving average are means (*n* = 24). (C, D) Distributions and bar graphs of the period lengths of bioluminescences in MEFs from *CKIδ* and *CK2α* mutant chimeric mice. Arrowheads show the mean. Error bars are SD. Statistical differences were evaluated using one-way ANOVA followed by Bonferroni post hoc test. ***P<0.001.

To investigate whether the abnormal period-length observed in *in vitro* differentiation culture of mutant ES cells recapitulates the characters of circadian clock developed *in vivo*, we generated chimeric mice by injecting WT and mutant ES cells into BDF1 blastocysts. Since *CKIδ* knock-out mice were perinatal lethal and *CK2α* knock-out mice were embryonic lethal, we prepared MEFs from E13.5 chimera embryos instead of mice **(** **Figure 5A** **)**. In these MEFs, we were able to specifically monitor bioluminescence originated from ES cell lines, because host embryos are incapable of expressing the bioluminescence marker. In addition, it has been revealed that fibroblast oscillators are not influenced by the circadian properties of neighboring cells [28]. Therefore we analyzed the bioluminescence oscillation from the mixture of MEFs composed of host-derived and ES cell-derived MEFs after three passages from embryo dissociation. PMT-based bioluminescence monitoring revealed that both *CKIδ* and *CK2α* mutant ES cell-derived MEFs displayed lengthened periods compared with WT ES cell-derived MEFs **(** **Figure 5B** **)**. Quantitative analysis confirmed significantly longer periods in *CKIδ* and *CK2α* mutant MEFs **(** **Figure 5C** **)**. *CKIδ* mutant MEFs showed 24.3 hour period, nearly two hours longer than WT ES MEFs **(** **Figure 5D** **upper and middle panels)**. On the other hand, *CK2α* mutant MEFs showed divergent period distribution **(** **Figure 5D** **lower panel)**. Since this *CK2α* mutant ES cell line abolished its gene expression, the observed phenotypes such as longer and variable periods may be characteristic of *CK2α* deficient cells. The reason for the divergent period-length of these cell was not uncertain; the loss of *CK2α* may have affected the circadian clock development in chimeric mice embryos with some different mechanisms from *CKIδ*. To our knowledge, this is the first direct genetic evidence showing the effect of *CK2α* deficiency on circadian clocks in mammalian peripheral cells.

## Discussion

It has been revealed that CKIδ plays a distinct role in mammalian circadian clock as a central kinase phosphorylating clock proteins [7]–[9]. In this study, the *in vitro* circadian clock formation assay revealed that *CKIδ* deficient ES cells developed circadian clock oscillation with a ∼ 3 hours longer period-length than WT, and these results are consistent with previously reported circadian phenotypes in MEFs and SCN from *CKIδ* knock-out mice [8], [18], [19]. Moreover, WT and revertant ES cells with normal *CKIδ* gene expression exhibited comparable circadian periods **(see** **Figure 4A, B and C** **)**, suggesting *in vitro* clock formation assay in ES cells faithfully reproduce the genetically determined circadian rhythms in mammals. In addition, the developed circadian rhythm from ES cells after *in vitro* differentiation culture exhibited temperature compensation (**see Figure S2**). These findings revealed that the *in vitro* circadian clock formation assay using ES cells exactly recapitulated the circadian clock phenotype (at least in cellular or tissue level) before making mice.

We also demonstrated that *CK2α* deficient ES cells developed at an approximately 2.5 hours longer period-length. The role of *CK2α* in circadian oscillation has been implicated from RNAi-mediated knock-down and/or chemical inhibition of CK2 [20], [29]–[31]. However, off-target effects cannot in general be excluded in RNAi and a chemical inhibitor. Although off-target effects could also accompany gene trap approach, isolation and characterization of revertant (Figure 2 and 4) would help evaluate this possibility. Furthermore, the gene knockout study of *CK2α* has been hampered due to embryonic lethality. Our approach of *in vitro* ES cell differentiation circumvents the problem of embryonic lethality and presents the first unequivocal evidence showing *CK2α* deficiency lengthens the period in mammalian cells including MEFs, establishing *CK2α* as an essential mammalian clock gene.

It should be noted that ES cell-based assay does not replace other assay systems such as RNAi, chemical library screening and knock-out mouse study. We rather consider that these assay systems are complementary to each other. RNAi and chemical library screening would be appropriate to study the effect of acute knock-down of target genes in a high-throughput manner. Some of the circadian phenotypes would be revealed only in whole animal studies using knock-out mice. In contrast, ES cell-based assays would reproduce developmental process to generate circadian clock in tissue culture and allow for in-depth analysis of circadian clock formation in a time-dependent manner. The ES cell-based phenotype assay would provide an alternative approach to study gene functions *in vitro*.

### Conclusion

Taken together, our results suggest that the ES cell-based *in vitro* circadian clock recapitulation assay is a powerful tool to evaluate genetic effects, especially when gene mutation causes embryonic lethality. Using this assay, we revealed that CK2 is an essential kinase to maintain the intact circadian period-length. Furthermore, this assay can also be utilized for ES cells-based circadian genetic screening complementary to an RNAi screening.

## Supporting Information

Figure S1 The reproducibility of the development of circadian rhythms. Representative raw bioluminescence traces of *in vitro* 28-day differentiated wild type ES cells.
(TIFF)

Figure S2 Temperature compensation of the period length from wild type ES cells carrying the *Bmal1:luc* reporter after *in vitro* 28-day differentiation. The graph indicates the mean ± SD. The lines indicate estimation from the equation *y* = 14.92+0.21*x* (peak) or *y* = 16.74+0.15*x* (trough). The Q10 values between 27°C and 37°C calculated from the equation are 0.907 (peak) or 0.932 (trough). The period length of the observed bioluminescence rhythms was well compensated or slightly over-compensated in the range of 29°C–35°C.
(TIFF)

Figure S3 Heat map plots of bioluminescence intensity of *in vitro* differentiated *Bmal1:luc* ES cells. Each horizontal line represents ES cells from a single EB differentiated *in vitro* for 7, 15, 24 and 28 days. Values above and below the mean are shown in red and green, respectively.
(TIFF)

## References

[1] Lowrey PL, Takahashi JS (2011) Genetics of circadian rhythms in Mammalian model organisms. Adv Genet 74: 175–230. 21924978 10.1016/B978-0-12-387690-4.00006-4 PMC3709251

[2] Bass J (2012) Circadian topology of metabolism. Nature 491: 348–356. 23151577 10.1038/nature11704

[3] Masri S, Zocchi L, Katada S, Mora E, Sassone-Corsi P (2012) The circadian clock transcriptional complex: metabolic feedback intersects with epigenetic control. Ann N Y Acad Sci 1264: 103–109. 22834651 10.1111/j.1749-6632.2012.06649.x PMC3464365

[4] Schibler U, Naef F (2005) Cellular oscillators: rhythmic gene expression and metabolism. Curr Opin Cell Biol 17: 223–229. 15780601 10.1016/j.ceb.2005.01.007

[5] Reppert SM, Weaver DR (2002) Coordination of circadian timing in mammals. Nature 418: 935–941. 12198538 10.1038/nature00965

[6] Preitner N, Damiola F, Lopez-Molina L, Zakany J, Duboule D, et al (2002) The orphan nuclear receptor REV-ERBalpha controls circadian transcription within the positive limb of the mammalian circadian oscillator. Cell 110: 251–260. 12150932 10.1016/s0092-8674(02)00825-5

[7] Eide EJ, Kang H, Crapo S, Gallego M, Virshup DM (2005) Casein kinase I in the mammalian circadian clock. Methods Enzymol 393: 408–418. 15817302 10.1016/S0076-6879(05)93019-X PMC1513158

[8] Lee H, Chen R, Lee Y, Yoo S, Lee C (2009) Essential roles of CKIdelta and CKIepsilon in the mammalian circadian clock. Proc Natl Acad Sci U S A 106: 21359–21364. 19948962 10.1073/pnas.0906651106 PMC2795500

[9] Xu Y, Padiath QS, Shapiro RE, Jones CR, Wu SC, et al (2005) Functional consequences of a CKIdelta mutation causing familial advanced sleep phase syndrome. Nature 434: 640–644. 15800623 10.1038/nature03453

[10] Isojima Y, Nakajima M, Ukai H, Fujishima H, Yamada RG, et al (2009) CKIepsilon/delta-dependent phosphorylation is a temperature-insensitive, period-determining process in the mammalian circadian clock. Proc Natl Acad Sci U S A 106: 15744–15749. 19805222 10.1073/pnas.0908733106 PMC2736905

[11] Reppert SM, Schwartz WJ (1983) Maternal coordination of the fetal biological clock in utero. Science 220: 969–971. 6844923 10.1126/science.6844923

[12] Sumova A, Bendova Z, Sladek M, El-Hennamy R, Laurinova K, et al (2006) Setting the biological time in central and peripheral clocks during ontogenesis. FEBS Lett 580: 2836–2842. 16563387 10.1016/j.febslet.2006.03.023

[13] Yagita K, Horie K, Koinuma S, Nakamura W, Yamanaka I, et al (2010) Development of the circadian oscillator during differentiation of mouse embryonic stem cells in vitro. Proc Natl Acad Sci U S A 107: 3846–3851. 20133594 10.1073/pnas.0913256107 PMC2840478

[14] Kowalska E, Moriggi E, Bauer C, Dibner C, Brown SA (2010) The circadian clock starts ticking at a developmentally early stage. J Biol Rhythms 25: 442–449. 21135160 10.1177/0748730410385281

[15] Vitaterna MH, King DP, Chang AM, Kornhauser JM, Lowrey PL, et al (1994) Mutagenesis and mapping of a mouse gene, Clock, essential for circadian behavior. Science 264: 719–725. 8171325 10.1126/science.8171325 PMC3839659

[16] King DP, Zhao Y, Sangoram AM, Wilsbacher LD, Tanaka M, et al (1997) Positional cloning of the mouse circadian clock gene. Cell 89: 641–653. 9160755 10.1016/s0092-8674(00)80245-7 PMC3815553

[17] Horie K, Kokubu C, Yoshida J, Akagi K, Isotani A, et al (2011) A homozygous mutant embryonic stem cell bank applicable for phenotype-driven genetic screening. Nat Methods 8: 1071–1077. 22020066 10.1038/nmeth.1739

[18] Etchegaray JP, Machida KK, Noton E, Constance CM, Dallmann R, et al (2009) Casein kinase 1 delta regulates the pace of the mammalian circadian clock. Mol Cell Biol 29: 3853–3866. 19414593 10.1128/MCB.00338-09 PMC2704743

[19] Etchegaray JP, Yu EA, Indic P, Dallmann R, Weaver DR (2010) Casein kinase 1 delta (CK1delta) regulates period length of the mouse suprachiasmatic circadian clock in vitro. PLoS One 5: e10303. 20421981 10.1371/journal.pone.0010303 PMC2858662

[20] Maier B, Wendt S, Vanselow JT, Wallach T, Reischl S, et al (2009) A large-scale functional RNAi screen reveals a role for CK2 in the mammalian circadian clock. Genes Dev 23: 708–718. 19299560 10.1101/gad.512209 PMC2661607

[21] Smith EM, Lin JM, Meissner RA, Allada R (2008) Dominant-negative CK2alpha induces potent effects on circadian rhythmicity. PLoS Genet 4: e12. 18208335 10.1371/journal.pgen.0040012 PMC2211540

[22] Mehra A, Shi M, Baker CL, Colot HV, Loros JJ, et al (2009) A role for casein kinase 2 in the mechanism underlying circadian temperature compensation. Cell 137: 749–760. 19450520 10.1016/j.cell.2009.03.019 PMC2718715

[23] Urasaki A, Morvan G, Kawakami K (2006) Functional dissection of the Tol2 transposable element identified the minimal cis-sequence and a highly repetitive sequence in the subterminal region essential for transposition. Genetics 174: 639–649. 16959904 10.1534/genetics.106.060244 PMC1602067

[24] Kiyohara YB, Tagao S, Tamanini F, Morita A, Sugisawa Y, et al (2006) The BMAL1 C terminus regulates the circadian transcription feedback loop. Proc Natl Acad Sci U S A 103: 10074–10079. 16777965 10.1073/pnas.0601416103 PMC1502508

[25] Okamoto K, Onai K, Ishiura M (2005) RAP, an integrated program for monitoring bioluminescence and analyzing circadian rhythms in real time. Anal Biochem 340: 193–200. 15840491 10.1016/j.ab.2004.11.007

[26] Liu AC, Welsh DK, Ko CH, Tran HG, Zhang EE, et al (2007) Intercellular coupling confers robustness against mutations in the SCN circadian clock network. Cell 129: 605–616. 17482552 10.1016/j.cell.2007.02.047 PMC3749832

[27] Eisen MB, Spellman PT, Brown PO, Botstein D (1998) Cluster analysis and display of genome-wide expression patterns. Proc Natl Acad Sci U S A 95: 14863–14868. 9843981 10.1073/pnas.95.25.14863 PMC24541

[28] Nagoshi E, Saini C, Bauer C, Laroche T, Naef F, et al (2004) Circadian gene expression in individual fibroblasts: cell-autonomous and self-sustained oscillators pass time to daughter cells. Cell 119: 693–705. 15550250 10.1016/j.cell.2004.11.015

[29] Hirota T, Kay SA (2009) High-throughput screening and chemical biology: new approaches for understanding circadian clock mechanisms. Chem Biol 16: 921–927. 19778719 10.1016/j.chembiol.2009.09.002 PMC2835411

[30] Tsuchiya Y, Akashi M, Matsuda M, Goto K, Miyata Y, et al (2009) Involvement of the protein kinase CK2 in the regulation of mammalian circadian rhythms. Sci Signal 2: ra26. 19491384 10.1126/scisignal.2000305

[31] Yagita K, Yamanaka I, Koinuma S, Shigeyoshi Y, Uchiyama Y (2009) Mini screening of kinase inhibitors affecting period-length of mammalian cellular circadian clock. Acta Histochem Cytochem 42: 89–93. 19617956 10.1267/ahc.09015 PMC2711227

