# Supplementary figures and images for: An In Vitro ES Cell-Based Clock Recapitulation Assay Model Identifies CK2α as an Endogenous Clock Regulator
Source: PLoS One. 2013 Jun 28;8(6):e67241. doi: 10.1371/journal.pone.0067241 (PMC3696008; doi:10.1371/journal.pone.0067241)

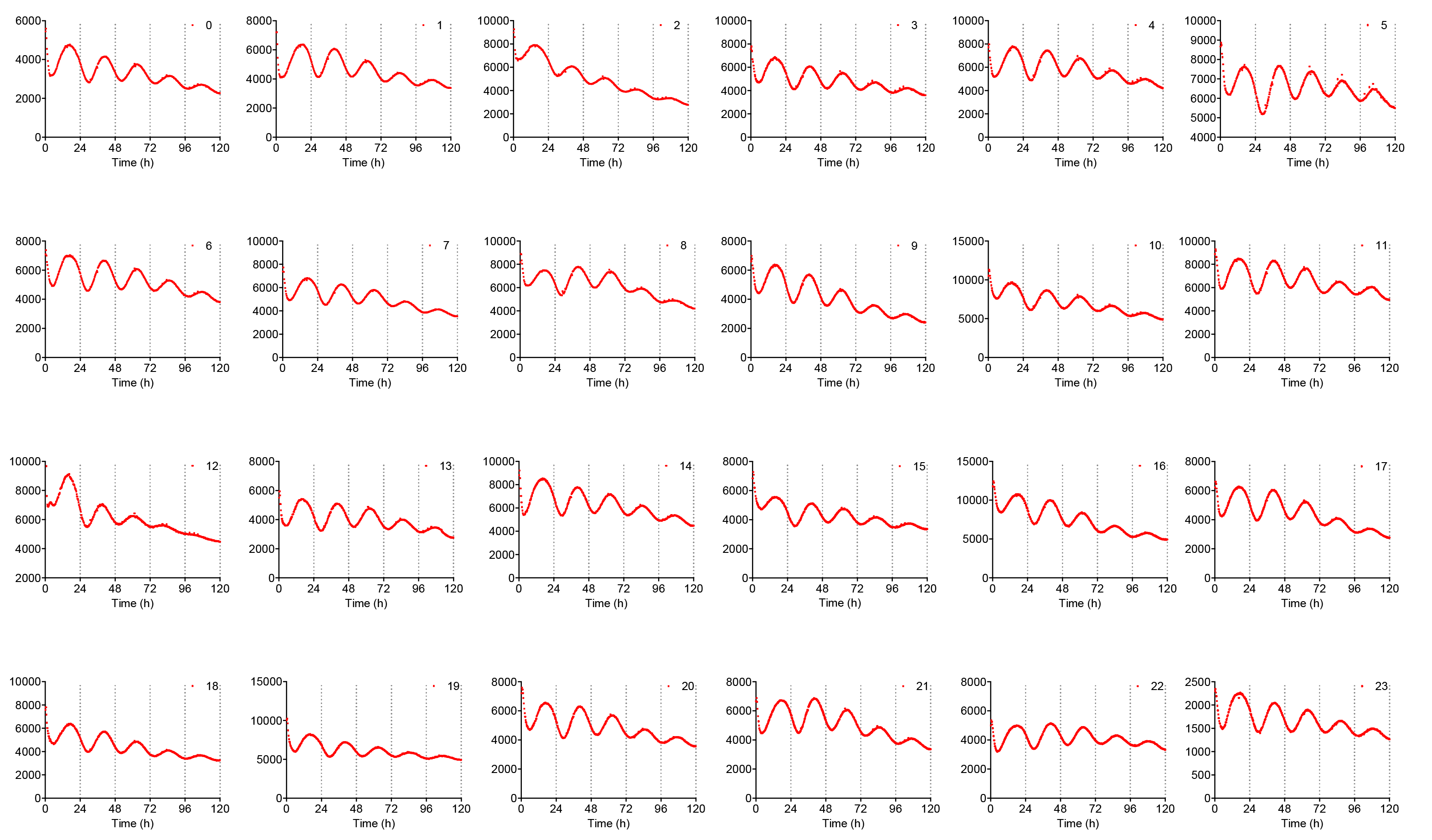

Supplement: Figure S1 — The reproducibility of the development of circadian rhythms. Representative raw bioluminescence traces of in vitro 28-day differentiated wild type ES cells. (TIFF) [file pone.0067241.s001.tiff]

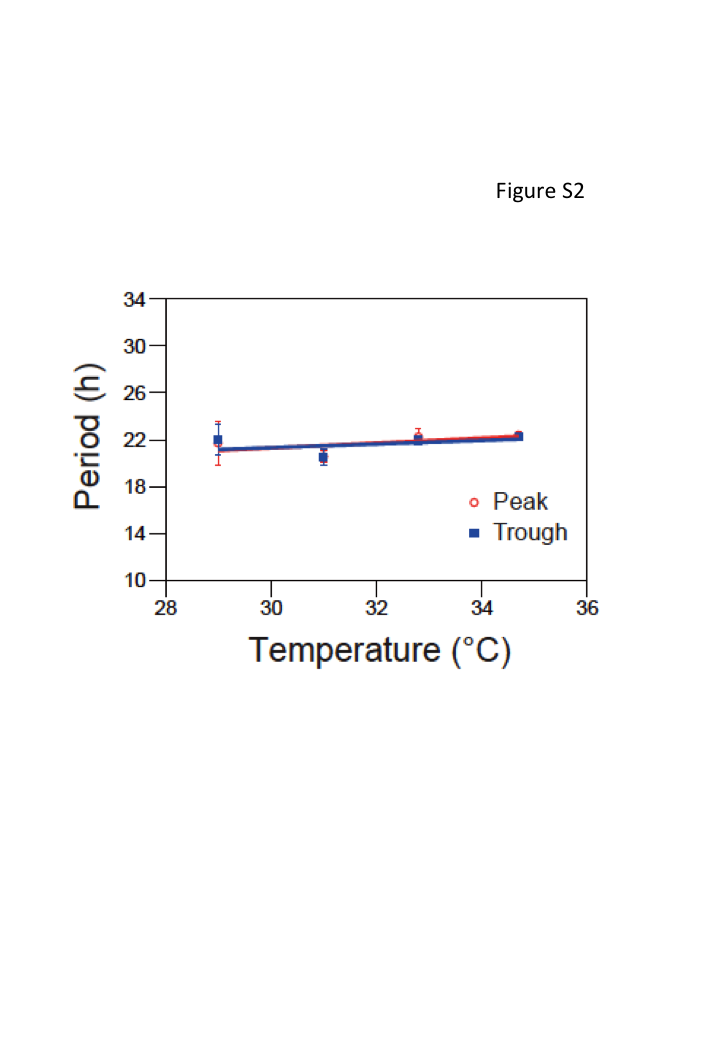

Supplement: Figure S2 — Temperature compensation of the period length from wild type ES cells carrying the Bmal1:luc reporter after in vitro 28-day differentiation. The graph indicates the mean ± SD. The lines indicate estimation from the equation y = 14.92+0.21x (peak) or y = 16.74+0.15x (trough). The Q10 values between 27°C and 37°C calculated from the equation are 0.907 (peak) or 0.932 (trough). The period length of the observed bioluminescence rhythms was well compensated or slightly over-compensated in the range of 29°C–35°C. (TIFF) [file pone.0067241.s002.tiff]

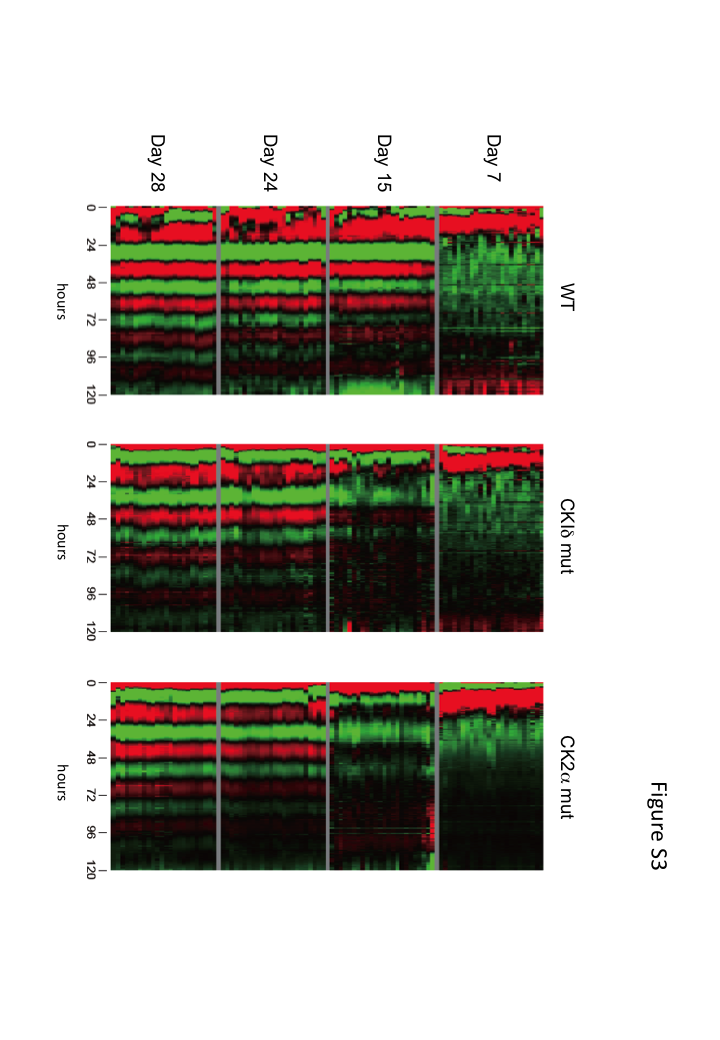

Supplement: Figure S3 — Heat map plots of bioluminescence intensity of in vitro differentiated Bmal1:luc ES cells. Each horizontal line represents ES cells from a single EB differentiated in vitro for 7, 15, 24 and 28 days. Values above and below the mean are shown in red and green, respectively. (TIFF) [file pone.0067241.s003.tiff]
